# Day-to-Day Test-Retest Reliability of EEG Profiles in Children With Autism Spectrum Disorder and Typical Development

**DOI:** 10.3389/fnint.2020.00021

**Published:** 2020-04-30

**Authors:** April R. Levin, Adam J. Naples, Aaron Wolfe Scheffler, Sara J. Webb, Frederick Shic, Catherine A. Sugar, Michael Murias, Raphael A. Bernier, Katarzyna Chawarska, Geraldine Dawson, Susan Faja, Shafali Jeste, Charles A. Nelson, James C. McPartland, Damla Şentürk

**Affiliations:** ^1^Department of Neurology, Boston Children’s Hospital, Harvard Medical School, Boston, MA, United States; ^2^Child Study Center, School of Medicine, Yale University, New Haven, CT, United States; ^3^Department of Epidemiology and Biostatistics, University of California, San Francisco, San Francisco, CA, United States; ^4^Center for Child Health, Behavior, and Development, Seattle Children’s Research Institute, Seattle, WA, United States; ^5^Department of Psychiatry and Behavioral Sciences, University of Washington, Seattle, WA, United States; ^6^Department of Biostatistics, University of California, Los Angeles, Los Angeles, CA, United States; ^7^Department of Psychiatry and Biobehavioral Sciences, University of California, Los Angeles, Los Angeles, CA, United States; ^8^Institute for Innovations in Developmental Sciences, Northwestern University, Chicago, IL, United States; ^9^Duke Institute for Brain Sciences, Duke University, Durham, NC, United States; ^10^Duke Center for Autism and Brain Development, Duke University, Durham, NC, United States; ^11^Department of Psychiatry and Behavioral Sciences, Duke University, Durham, NC, United States; ^12^Laboratories of Cognitive Neuroscience, Division of Developmental Medicine, Boston Children’s Hospital, Harvard Medical School, Boston, MA, United States

**Keywords:** EEG, autism, autism spectrum disorder, test-retest, power, FOOOF, reliability

## Abstract

Biomarker development is currently a high priority in neurodevelopmental disorder research. For many types of biomarkers (particularly biomarkers of diagnosis), reliability over short periods is critically important. In the field of autism spectrum disorder (ASD), resting electroencephalography (EEG) power spectral densities (PSD) are well-studied for their potential as biomarkers. Classically, such data have been decomposed into pre-specified frequency bands (e.g., delta, theta, alpha, beta, and gamma). Recent technical advances, such as the Fitting Oscillations and One-Over-F (FOOOF) algorithm, allow for targeted characterization of the features that naturally emerge within an EEG PSD, permitting a more detailed characterization of the frequency band-agnostic shape of each individual’s EEG PSD. Here, using two resting EEGs collected a median of 6 days apart from 22 children with ASD and 25 typically developing (TD) controls during the Feasibility Visit of the Autism Biomarkers Consortium for Clinical Trials, we estimate test-retest reliability based on the characterization of the PSD shape in two ways: (1) Using the FOOOF algorithm we estimate six parameters (offset, slope, number of peaks, and amplitude, center frequency and bandwidth of the largest alpha peak) that characterize the shape of the EEG PSD; and (2) using nonparametric functional data analyses, we decompose the shape of the EEG PSD into a reduced set of basis functions that characterize individual power spectrum shapes. We show that individuals exhibit idiosyncratic PSD signatures that are stable over recording sessions using both characterizations. Our data show that EEG activity from a brief 2-min recording provides an efficient window into characterizing brain activity at the single-subject level with desirable psychometric characteristics that persist across different analytical decomposition methods. This is a necessary step towards analytical validation of biomarkers based on the EEG PSD and provides insights into parameters of the PSD that offer short-term reliability (and thus promise as potential biomarkers of trait or diagnosis) vs. those that are more variable over the short term (and thus may index state or other rapidly dynamic measures of brain function). Future research should address the longer-term stability of the PSD, for purposes such as monitoring development or response to treatment.

## Introduction

The development of translational biomarkers is a crucial step towards clinical trial readiness for neurodevelopmental disorders such as autism spectrum disorder (ASD; Sahin et al., 2018). The recent failure of several promising clinical trials (Krueger et al., 2017; Berry-Kravis et al., 2018) underscores the importance of biomarker development, and the need for a range of biomarkers serving a range of purposes. For example, a diagnostic biomarker can confirm the presence or absence of a disorder, or identify individuals with a biologically-defined subtype thereof (FDA-NIH Biomarker Working Group, 2016), to guide patient selection for clinical trials. A monitoring biomarker can serially assess the status of a disorder (FDA-NIH Biomarker Working Group, 2016), and thus measure the response to medical therapies or other exposures. The ideal properties of a given biomarker thus depend largely on its context of use. For example, a diagnostic biomarker should not change significantly over a given time window if the biology of the disorder it is indexing has not changed. On the other hand, a monitoring biomarker should change over time in a manner that reflects the biological impact of a medical treatment.

One of the most promising imaging tools for biomarker development in neurodevelopmental disorders is electroencephalography (EEG). EEG is an index of the neural networks that bridge genotype to phenotype across a variety of ages, disorders, and species, and thus offers substantial promise for the development of scalable biomarkers that are relevant to the brain mechanisms underlying ASD (Port et al., 2014; Jeste et al., 2015). Within EEG, the power spectral density (PSD), which represents the contributions of oscillations at various frequencies to the EEG, offers both diagnostic and monitoring potential. For example, among children with ASD compared to typical development, there is evidence that the resting PSD shows (at a group level) higher power in the low (delta, theta) and high (beta, gamma) frequency bands and lower power in the middle (alpha) frequency bands (Wang et al., 2013). This suggests the potential utility of some aspects of the PSD as a diagnostic biomarker for autism. Moreover, EEG is a measure of cortical activity and is thus fundamentally dynamic; it changes throughout development, across awake and asleep states, and in response to pharmacological treatment. This suggests that there may be aspects of the PSD that offer potential in other categories of biomarker development (e.g., monitoring or response biomarkers).

Thus, to inform the development of biomarkers using EEG-based measures, it is necessary to evaluate the reliability of the PSD within an individual over brief time intervals, as well as across development and in response to various therapies. This is of particular importance in ASD, given the suggestion that intra-individual variability in brain activity may itself be an endophenotype of ASD (David et al., 2016). Different features of the PSD may exhibit different measurement properties, with some parameters reflecting more transient or “state-like” properties of brain activity and others reflecting more stable “trait-like” interindividual differences. To begin this process, in the present study, we focus on test-retest reliability of the PSD and specific parameters thereof over a short time window (median of 6 days) during which one would not expect significant changes in underlying diagnosis, developmental changes are minimal, no new treatments are given, and EEG is collected under identical conditions.

Prior studies in healthy adults have demonstrated good to excellent test-retest reliability for certain features of the PSD. EEG power for mid-range frequencies (theta, alpha, and beta, as opposed to delta and gamma; Ip et al., 2018) and relative power (as opposed to absolute power; Salinsky et al., 1991) have shown correlation coefficients >0.8 for EEG sessions a few weeks apart; this is in the range of test-retest correlations for commonly used tests of cognitive ability (Elliott, 2007; Canivez and Watkins, 1999). Methodological advances in EEG pre-processing, such as a robust reference to average and wavelet independent component analysis which act to attenuate the effects of data collection artifact, improve test-retest reliability in higher frequency bands such as beta and gamma (Suarez-Revelo et al., 2016). However, the reliability of these features in children with or without neurodevelopmental disabilities remains unmeasured.

Notably, traditional methods of characterizing the PSD rely on measuring power within a particular frequency band, which conflates important aspects of underlying EEG activity. First, the EEG PSD typically contains a series of periodic oscillations atop an aperiodic background activity in which the power decreases as frequency (f) increases, leading to a consistent 1/f^α^ distribution to the PSD, with the exponent α determining the slope of this background activity. This aperiodic activity, and the offset thereof, may reflect crucial mechanistic underpinnings of brain activity (He et al., 2010), such as tonic excitation/inhibition balance or total spiking activity of underlying neural populations respectively (Haller et al., 2018). The influence of this background activity on the measurement of oscillatory activity is partially (though not completely) eliminated using techniques such as normalization or log transform of the PSD. Second, *a priori* assumptions about the frequency bands wherein oscillations occur may compromise accurate measurement and fail to capture the meaningful variation of these oscillations. For example, averaging power in the predefined alpha range (e.g., 8–13 Hz) removes information about the peak alpha frequency in a given individual; however, the exact location of this alpha peak is well known to change with age and cognitive status (Angelakis et al., 2004; Grandy et al., 2013) and can even occur outside of the 8–13 Hz range. Because oscillations rarely span the exact range specified in a frequency band, their activity can be inadvertently included in neighboring frequency bands if they are wide or shifted. Finally, in cases where a periodic oscillation has a narrow bandwidth or is nonexistent with a prespecified frequency band, measurement of activity in that band will predominantly reflect the aperiodic activity. For these reasons, it is useful to characterize the EEG as a unique profile, with parameterization informed by the shape of each individual’s PSD rather than piecemeal averages across distinct frequency bands.

As of October 2019, ClinicalTrials.gov reported 315 currently recruiting studies collecting EEG data and of those 102 were recruiting pediatric populations. Given the extent of this ongoing research, addressing how best to characterize the profile of the EEG PSD and determine its reliability and stability over time, particularly in clinical and developmental populations, is both important and timely. Such work forms an important foundation on which to base future research, and provides critical information to contextualize current findings.

In this study, we, therefore, explore the test-retest reliability of the profile of the EEG PSD in children with ASD and typical development (TD) over EEG recordings conducted within a short (~6 days) time-span. We applied two approaches to characterizing the profile of the PSD: (1) parametric model-based decomposition of the PSD into offset, slope, and oscillatory peaks using the Fitting Oscillations and One-Over-F (FOOOF) algorithm (Haller et al., 2018); and (2) nonparametric functional data analysis, which identifies a small set of principal component functions that combine to describe the shape of the power spectrum. We hypothesized that these complementary approaches would exhibit high levels of short-term test-retest reliability. In this way, we demonstrate the utility of resting EEG PSD shape, and some specific parameters thereof, as stable biomarkers of cortical activity over short time windows.

## Materials and Methods

These data were collected as part of the ongoing Autism Biomarkers Consortium for Clinical Trials (ABC-CT^1^; McPartland, 2016). Details of the ABC-CT data acquisition are reported elsewhere (Webb et al., 2019; McPartland et al., 2020). The objective of the ABC-CT is to evaluate a set of electrophysiological (EEG), eye-tracking, and behavioral measures for use in clinical trials for ASD. The ABC-CT began with a “Feasibility Study,” which included the participants described below and involved two EEGs separated by a short window of time (median 6 days) as described below. The ABC-CT then moved on to the “Main Study,” which included a larger number of participants, with EEGs separated by longer windows of time (6 weeks, and then 6 months). Only the data from the “Feasibility Study” is included here, as the focus of this manuscript is on the shorter-term test-retest reliability of the EEG PSD; this type of information (two EEGs separated by a few days) was not collected in the “Main Study.” This study was carried out following the recommendations of the central Institutional Review Board at Yale University, with written informed consent from a parent or legal guardian and assent from each child before their participation in the study.

### Participants

Fifty-one participants (25 with ASD, 26 with TD), were enrolled in the feasibility phase of the ABC-CT. Inclusion criteria included age 4–11 years, IQ 50–150 (as assessed by the Differential Ability Scales–2nd Edition), and participant and their parent/guardian must be English speaking. Exclusion criteria included a known genetic or neurological syndrome, metabolic disorder, mitochondrial dysfunction, significant sensory and/or motor impairment not correctable by a hearing aid or glasses/contact lenses, and history of significant prenatal/perinatal/birth injury, neonatal brain damage, or epilepsy. All participants (and at least one biological parent, if accompanying the child to the visit) were required to participate in a blood draw. Medication was not exclusionary, but participants were required to have been stable for 8 weeks on a current medication regimen. Additionally, environmental circumstances likely to account for ASD (e.g., severe nutritional or psychological deprivation) were exclusionary in the ASD group. In the TD group, additional exclusionary criteria included an active psychiatric disorder, a historical diagnosis of ASD, or a sibling with ASD.

Group characteristics are presented in Table 1. Groups differed significantly on age (*t*_(45)_ = 2.3, *p* = 0.025) and IQ (*t*_(45)_ = 4.6, *p* < 0.001). One participant with ASD and 3 participants with TD were left-handed. The “Feasibility Study Visit” consisted of two EEGs on two separate days (termed here “Day 1” and “Day 2”), separated by a short window of time (range 1–22 days, median 6 days) during this phase. Participants were characterized using rigorous autism diagnostic standardized measures [Autism Diagnostic Observation Schedule, 2nd edition (ADOS-2; Lord et al., 2001), Autism Diagnostic Interview-Revised (ADI-R; Lord et al., 1994), and Diagnostic and Statistical Manual of Mental Disorders (DSM-5) criteria (American Psychiatric Association, 2013)] by research-reliable clinicians (Webb et al., 2019), and cognitive measures [Differential Ability Scales 2nd edition (DAS-II; Elliott, 2007)].

**Table 1:** Participant sex, age, and IQ by diagnostic group.

Group	*N* (N female)	Mean Age (Y)	Min. Age (Y)	Max. Age (Y)	Mean IQ (SD)
ASD	24 (5)	8.0*	4.42	11.4	93 (18.2)*
TD	26 (9)	6.6	4.01	11.4	114 (9.4)

*Indicates measures that differ by group, as described in the text.

### EEG Protocol

In the feasibility phase of the ABC-CT, EEG acquisition included six paradigms (Webb et al., 2019), with “Resting EEG eyes open during calm viewing” of silent, chromatic digital videos (similar to screensavers) collected twice on two separate days. Video stimuli consisted of six 30 s non-social abstract videos purchased from Shutterstock, which were presented to the participant in random order in three blocks of 1 min on each day (Webb et al., 2018). The videos were played forward for 15 s and then reversed for the following 15 s. To allow for counterbalancing of the methods used in the ABC-CT (Eye Tracking and EEG), at screening, participants were stratified based on variables that could be assessed by phone to include group (ASD/TD), biological sex (male/female), age (split at 8 years 6 months), and cognitive ability (ASD only, assessed in person by a trained clinician at first visit). Half of the participants received eye-tracking first at each visit and the other half received EEG first.

Data were collected at five different sites. All sites had a high-density EEG acquisition system (Philips Neuro, Eugene, OR, USA), including either Net Amps 300 (Boston Children’s Hospital, University of California Los Angeles, University of Washington, and Yale University) or Net Amps 400 amplifiers (Duke University). All sites used the 128 electrode HydroCel Geodesic Sensor Nets, applied according to Philips Neuro/Electrical Geodesics, Inc. standards. Four of the five sites removed electrodes 125–128, which are positioned on the participant’s face, from the EEG caps to the tolerability of wearing the cap. Appropriate EEG acquisition protocols and software (500 Hz sampling rate, MFF file format, onset recording of amplifier and impedance calibrations) were provided to each site. EPrime 2.0 (Psychological Software Tools, Sharpsburg, PA, USA) was used for experimental control. The coordinating site reviewed and provided feedback on the net application, adherence to administration protocol, and data quality for every session. Sites conducted regular monthly checks of equipment function.

One participant with ASD refused to wear the net; EEG data was therefore available on 24 ASD and 26 TD participants. After the preprocessing described below, EEG from one additional ASD participant was excluded from the parametric and nonparametric data analyses due to having a substantially lower number of observed segments than the rest of the sample (61 segments vs. an average of 91 segments) and only 1 day of EEG recording. Thus, in total, there was usable data on at least 1 day from 23 ASD and 26 TD participants (N: Duke_ASD_ = 4; Duke_TD_ = 5; BCH_TD_ = 5; BCH_ASD_ = 5; Yale_TD_ = 5; Yale_ASD_ = 5; UW_TD_ = 5; UW_ASD_ = 5; UCLA_TD_ = 6; UCLA_ASD_ = 5). Data on one ASD and one TD participant were recorded only on day 1. There was thus usable data on both days from 22 ASD and 25 TD participants (of note, Table 1 includes data on all participants who had EEG data available on at least 1 day, and not just those who contributed 2 days of EEG; this is because the mixed-effects models described below can still make use of the data from participants who contributed just 1 day of EEG).

### Preprocessing of the EEG

Processing of the raw EEG data was done using the Harvard Automated Processing Pipeline for Electroencephalography (HAPPE; Gabard-Durnam et al., 2018) embedded within the Batch EEG Automated Processing Platform (BEAPP; Levin et al., 2018). In brief, data were 1 Hz high pass and 100 Hz low pass filtered, downsampled to 250 Hz, and run through the HAPPE module including a selection of 18 channels corresponding to the 10-20 system channels (excluding Cz, as data were originally collected in reference to Cz), 60 Hz electrical line noise removal, bad channel rejection, wavelet-enhanced thresholding, independent component analysis with automated component rejection (Winkler et al., 2011, 2014), automated segment rejection, interpolation of bad channels, and re-referencing to average (Of note, the selection of 18 channels from the full 128-channels is necessary to generate a robust signal decomposition using independent component analysis, given the short length of the EEG recording. Details of how to determine an appropriate number of channels included in an independent component analysis decomposition are provided elsewhere; Gabard-Durnam et al., 2018; Levin et al., 2018). Data were then segmented into two-second segments, and the PSD was calculated *via* multitaper spectral analysis (Thomson, 1982; Babadi and Brown, 2014) using three tapers. The PSD was estimated for each participant and electrode by averaging the PSDs of artifact-free segments. Scalp-wide spectral densities were obtained by averaging spectral densities across the 18 electrodes for each subject on each day. Parametric analyses were based on absolute power, whereas nonparametric analyses were based on relative power.

### Parametric Decomposition of Periodic and Aperiodic Activity

**Figure 1:**
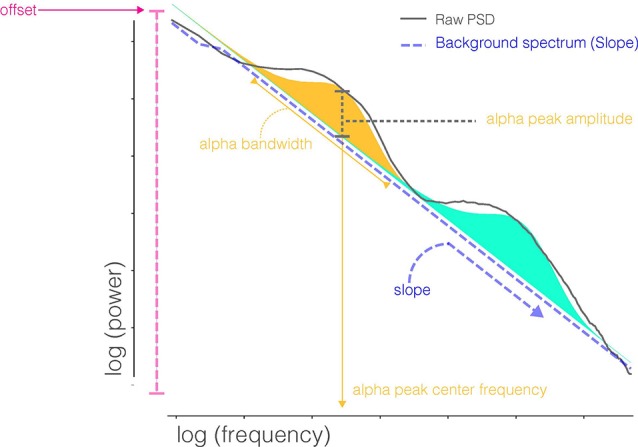
Parameters extracted from FOOOF decomposition of the power spectral densities (PSD). FOOOF models individual oscillatory peaks atop the PSD and estimates the slope and offset of aperiodic activity below those peaks. Shaded regions (blue and orange) indicate distinct oscillatory peaks identified by model fitting.

In order to characterize periodic and aperiodic features of the PSD profile, we used the Fitting Oscillations and One-Over-F (FOOOF) algorithm (Haller et al., 2018). The algorithm operates by removing an aperiodic slope (Figure 1) from the absolute PSD in the semilog-power space (linear frequencies and logged power), which is fully characterized by offset and slope terms. After removing the aperiodic component, the spectral density contains periodic oscillatory peaks that are modeled as a finite sum of Gaussians. Each Gaussian peak is defined by its amplitude, center frequency, and bandwidth (defined as two standard deviations of the fitted Gaussian). Thus, the PSD profile, including both the aperiodic background and periodic oscillations, can be fully parameterized by the following parameters: offset, slope, number of peaks (Gaussians), and the center frequency, amplitude, and bandwidth for each peak. These scalar features are then available for analysis across recording sessions using standard statistical techniques. The FOOOF model parameters were chosen by visually inspecting model fit across a range of parameters, blind to participant group and recording session, and selecting those which best captured oscillatory peaks across all of the recordings. A single parameter set was selected for all recordings. Specifically, the bandwidth of oscillatory peaks ranged between 1 and 10 Hz, and the minimum peak height (to be included in the fit) was 1.85 standard deviations above the aperiodic background activity.

Since the number of total peaks identified on each spectral density varied across subjects and days, for comparison purposes across consecutive days we first considered the agreement of the location [in terms of frequency band, i.e., delta (2–4 Hz), theta (4–6 Hz), low alpha (6–9 Hz), high alpha (9–13 Hz), beta (13–30 Hz), and gamma (30–55 Hz)] of the peak with the largest amplitude between days. For comparison of the largest peak features (center frequency, amplitude, and bandwidth), we then considered the largest peak in the entire alpha band for stability of results and ease of comparison between diagnostic groups. This allowed characterization of each scalp-wide spectral density by six FOOOF parameters: offset, slope, number of peaks, and (for the largest peak in the alpha range) center frequency, amplitude, and bandwidth. The agreement of these six FOOOF parameters across the 2 days for each diagnostic group was evaluated using the intraclass correlation coefficient (the ratio of between-person variance to total variance; ICC; Donner and Koval, 1980). Age-adjusted and IQ-adjusted ICCs are also presented, by adding these variables as predictors in the mixed-effects model. ICC values less than 0.40 are considered poor, between 0.40 and 0.59 fair, between 0.60 and 0.74 good, and between 0.75 and 1.00 excellent (Cicchetti, 1994). For all reported ICC values, bootstrap based on resampling subjects with replacement was used for forming percentile confidence intervals (CIs). Bootstrap methods yield more reliable inference in small samples (bootstrap CIs were based on 200 resampled data sets).

### Nonparametric Analysis of the Relative Spectral Density *via* Functional Data Analysis

Scalp-wide relative spectral densities were obtained by averaging relative spectral densities across electrodes for each subject observed on each day. The agreement in relative spectral density across days for both electrode-specific and scalp-wide relative spectral densities was computed by functional ICC within each diagnostic group. Since a trend of lower functional ICC was observed for the most peripheral electrodes [electrodes 9 (FP2), 22 (FP1), 45 (T3), 70 (O1), 83 (O2) and 108 (T4)] across diagnostic groups, a sensitivity analysis was also run through the functional ICC of the scalp-wide relative spectral densities excluding these six electrodes. Computation of functional ICC follows a functional ANOVA decomposition of the data within each diagnostic group with days as the within-subject factor. Functional ICC is the functional analog of the intra-class correlation in standard mixed-effects models. It corresponds to the ratio of the between-subject variability to total variance (between + within) similar to ICC but estimates variance parameters using functional data analysis techniques. Hence it can be interpreted as the intra-subject correlation of the entire relative spectral density across days, as opposed to the ICC for the FOOOF parameters which refer to the stability of certain features of the spectral density (but not the spectral density in its entirety). The functional ANOVA model is fit using a multilevel functional principal component decomposition (Di et al., 2009) which entails estimation of the subject- and day-level eigenvalues and eigenfunctions that enrich interpretations by allowing us to connect the nonparametric functional data analysis to results from the parametric analysis *via* FOOOF. For all reported functional ICC values, bootstrap percentile CIs were formed based on 200 resampled data sets based on resampling from subjects with replacement.

## Results

Age, sex, and IQ for study participants are in Table 1.

**Figure 2:**
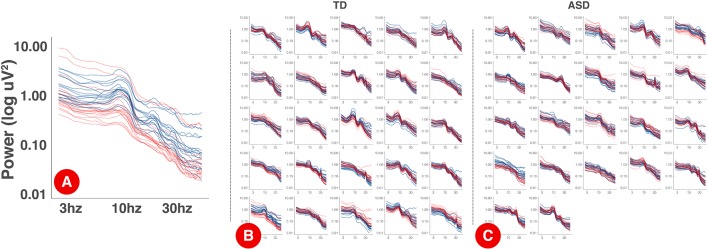
PSDs for each session by participant. Panel **(A)** displays an expanded, single participant, PSD with the log-10 axis labels. Each electrode is a single line. Day one PSDs are shown in blue and day 2 PSDs are shown in red. Panels **(B)** and **(C)** show individual PSDs for TD **(B)** and ASD **(C)** participants. Each smaller figure is data from a single participant.

The power spectrum of each individual on day 1 and day 2 is plotted in Figure 2. Within participants, PSD shapes exhibit visual similarity across separate recording sessions.

Data quality metrics output from HAPPE (Gabard-Durnam et al., 2018) are described in Table 2. Overall, data quality was high across groups.

**Table 2:** Data quality measures, based on HAPPE metrics.

Group	Day	Good Channels (%)	# of EEG segments retained	Rejected components (%)	EEG variance retained (%)	Mean retained artifact probability	Median retained artifact probability
ASD	1	95.4 (3.4)	90.7 (1.8)	29 (11)	70.2 (17.1)	0.08 (0.03)	0.03 (0.02)
	2	95.9 (3.9)	90.7 (1.8)	30 (12)	70.6 (15.8)	0.08 (0.03)	0.02 (0.02)
TD	1	97.4 (3.8)	90.8 (1.7)	18 (10)	82.5 (13.2)	0.05 (0.02)	0.01 (0.01)
	2	97.1 (3.8)	90.9 (1.7)	19 (10)	80.2 (15.2)	0.06 (0.04)	0.02 (0.02)

*Data are reported as mean (SD). EEG segments are 2 s long*.

### Parametric Analysis of the Absolute Power Spectral Density *via* FOOOF

The location of the dominant peak (i.e., the peak with the greatest amplitude according to the FOOOF algorithm) from both days is provided in Table 3 for both diagnostic groups. The dominant peak occurred most frequently in the high alpha frequency band in the ASD group and low alpha frequency band in the TD group. Across days, while the dominant peak stayed within the alpha band (low and high alpha) mostly for the TD group, it stayed more broadly within the alpha-beta range in the ASD group.

**Table 3:** The location of the dominant peak in day 1 (rows) vs. day 2 (columns) among the TD and ASD groups.

Day 1/2	Low_Alpha	High_Alpha	Beta	Gamma
**TD**				
Low_Alpha	6	6	0	0
High_Alpha	5	3	0	1
Beta	1	1	0	0
Gamma	1	0	0	1
**ASD**				
Low_Alpha	2	2	1	0
High_Alpha	2	4	3	0
Beta	2	3	1	0
Gamma	0	1	1	0

*Values indicate the number of participants with a given combination of dominant peak locations across days*.

The estimated ICCs along with their bootstrap CIs for an agreement of the six FOOOF parameters derived from scalp-wide absolute PSD across the two experimental days are provided in Table 4 for both diagnostic groups. Among offset, slope, and number of peaks, offset yielded consistently fair agreement in both groups [TD 0.484 95% CI (0.004, 0.775); ASD 0.525 95% CI (0.167, 0.806)], with slope between the 2 days showing poor agreement in the TD group (0.284 95% CI (0, 0.674) but good agreement in the ASD group [0.699 95% CI (0.527, 0.815)]. Among the three FOOOF parameters describing the largest alpha peak, amplitude had the highest ICC in both groups [TD 0.862 95% CI (0.729, 0.939); ASD 0.828 95% CI (0.664, 0.926)], followed by center frequency [TD 0.700 95% CI (0.437, 0.862); ASD 0.619 95% CI (0.342, 0.852)], and bandwidth [TD 0.424 95% CI (0.028, 0.696); ASD 0.340 95% CI (0.034, 0.727)]. While the agreement of the largest alpha peak amplitude was high in both groups, agreement in the peak frequency was slightly higher in the TD group than the ASD group. In the sensitivity analysis, when the analysis was repeated on FOOOF parameters derived after the exclusion of the six peripheral electrodes, these results remained unchanged. Age-adjusted ICC values (Supplementary Table S1) are notable predominantly for a decrease in the ICC of the center frequency of the alpha peak (as compared to unadjusted ICC values). This decrease is larger in the TD group than the ASD group. The TD group also shows a decrease in ICC of the alpha bandwidth when adjusting for age. IQ-adjusted ICC values (Supplementary Table S2) remain largely unchanged from unadjusted ICC values.

**Table 4:** The estimated intraclass correlation coefficients (ICCs) and their 95% bootstrap CI for the six FOOOF parameters for each diagnostic group.

FOOOF Parameter	TD	ASD
Offset	0.484 (0.004, 0.775)	0.525 (0.167, 0.806)
Slope	0.284 (0, 0.674)	0.699 (0.527, 0.815)
Number of peaks	0.021 (0, 0.571)	0.226 (0.003, 0.609)
Largest alpha peak: Center	0.700 (0.437, 0.862)	0.619 (0.342, 0.852)
Frequency
Largest alpha peak: Amplitude	0.862 (0.729, 0.939)	0.828 (0.664, 0.926)
Largest alpha peak: Bandwidth	0.424 (0.028, 0.696)	0.340 (0.034, 0.727)

### Nonparametric Analysis of the Relative Power Spectral Density *via* Functional Data Analysis

**Figure 3:**
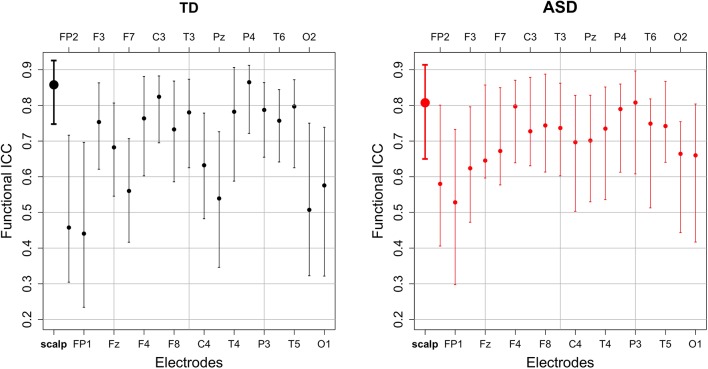
The estimated scalp-wide (bold) and electrode-specific functional intraclass correlations and their 95% bootstrap CI by diagnostic group.

The estimated functional ICC for the scalp-wide relative spectral density was excellent in both groups, though higher in the TD group than the ASD group [TD 0.858 95% CI (0.748, 0.926); ASD 0.807 95% CI (0.650, 0.914)]. The estimated functional ICC for each of the 18 electrodes and their 95% bootstrap CIs are shown by diagnostic group in Figure 3. While the average electrode-specific ICC in the TD group is approximately equal to that of the ASD group, there is greater variation in the functional ICC among electrodes in the TD group (both higher and lower values of the functional ICC) compared to the ASD group. In the sensitivity analysis, the estimated scalp-wide functional ICC for both diagnostic groups was slightly higher when the six peripheral electrodes are excluded [TD 0.874 95% CI (0.741, 0.931); ASD 0.815 95% CI (0.712, 0.913)], though the magnitude of difference between the two diagnostic groups was unchanged.

**Figure 4:**
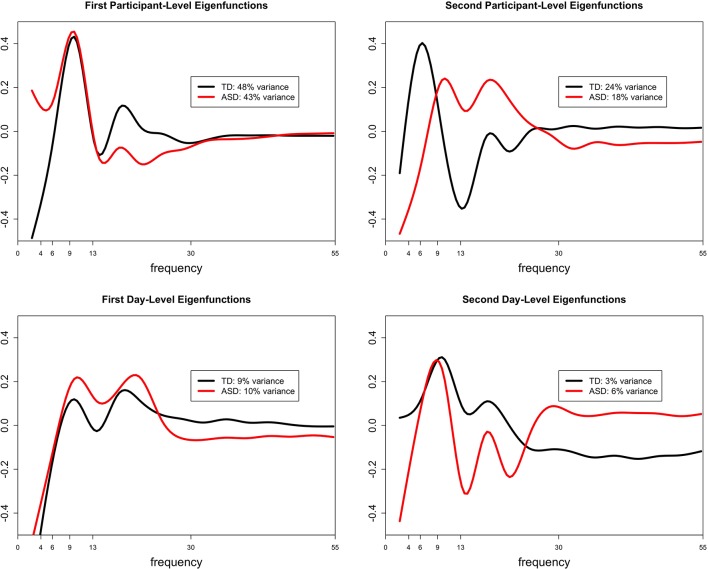
The estimated first and second leading eigenfunctions for the participant-level variation (top row) and day-level variation (bottom row) for each diagnostic group. The total variation explained by each component is included in the legend.

The functional ANOVA model captures individual deviations from the mean scalp-wide relative spectral density over the 2 days by partitioning the total variance into participant- and day-level variation. Participant-level variation captures the variation among participants whereas day-level variation captures the variation within a subject across days. Within each level of variation, ordered curves known as eigenfunctions identify which portions of the frequency domain account for the most variation by placing more magnitude at these locations. The two estimated leading participant- and day-level eigenfunctions for both diagnostic groups are shown in Figure 4. We restrict our discussion to the first two participant-level eigenfunctions, since combined they explain at least 60% of the total variation in both groups. We include the first 2 day-level eigenfunctions for completeness. The first participant-level eigenfunction for both groups displays that most variation in the data is explained by the variation in the amplitude of the alpha peak (with maximal variation at approximately 9 Hz), explaining similar total variation for the TD group (48% total variance explained) and the ASD group (43% total variance explained). While the first participant-level eigenfunction highlights variation in the amplitude of the largest peak, the second participant-level eigenfunction highlights the variation in the frequency (location) of the largest peak, where TD participants show the largest variation in the low and high alpha band (24% total variance explained) and ASD participants show it in high alpha and beta relative power (18% variance explained). These findings are consistent with the locations of the largest peak summarized in Table 3 across days for the two groups. While the first day-level eigenfunction highlights across day variability in alpha and beta relative power, the second eigenfunction highlights across day variability in the location of the largest peak (between high and low alpha for TD, and between high alpha and beta for ASD) similar to the second participant-level eigenfunction. The fact that most of the variation is explained by the participant-level eigenfunctions (compared to day-level eigenfunctions) supports our interpretation that most of the variation in the data is variation across subjects and there is less variability within a subject across days. Also, participants maintain stable alpha peaks across experimental days, both in terms of peak frequency and amplitude, consistent with the high ICCs reported in Table 4 for alpha peak amplitude and frequency in the two groups in the FOOOF analysis.

## Discussion

In this manuscript, we examine the test-retest reliability of the EEG power spectral density in children with ASD and TD. EEG power-based measures are currently being evaluated and employed as biomarkers in a variety of neurodevelopmental and psychiatric disorders, and analytical validation (including understanding the test-retest reliability of these measures) is an important early step in the biomarker development process (Micheel and Ball, 2010).

Overall, our findings demonstrate excellent test-retest reliability for scalp-wide EEG profiles. This high test-retest reliability reflects the overall stability of the EEG power spectrum over relatively short time windows (a few days). For the development of diagnostic biomarkers, this reliability is crucial—we would not expect the fundamental biology of the brain to change over several days without intervention, and therefore biomarkers indexing brain function for diagnostic purposes should not change significantly over this period.

On the other hand, there are scenarios in which we would not expect (or want) aspects of the EEG power spectrum to remain stable. For example, while markers of phenotypic traits may remain stable, markers of state and other modifiable factors (e.g., epileptiform activity) may vary over short periods. For example, changes in the emotional state during testing, and attention to the stimuli, may lead to changes in EEG power that reflect true physiologic changes in brain function over even short time windows. Similarly, scarce epileptiform activity may occur in some of a participant’s EEG recordings but not others. While the ABC-CT does not involve a specific intervention, this concept will become particularly relevant when treatments target a specific modifiable factor (e.g., psychotropic medications which may modify state; spike suppressing anti-epileptic medications which may modify epileptiform activity). Identifying the parameters of the EEG PSD that predominantly reflect stable factors (e.g., traits), and separately those that predominantly reflect modifiable factors (e.g., state, mood, attention, and epileptiform activity), while beyond the scope of the study described here, will allow us to harness the wealth of information available from EEG recordings to develop a range of biomarker types in future studies. This concept will be crucial for clinical trials as well. For example, monitoring biomarkers will ideally remain relatively stable when treatment is not given, but show a significant change in response to targeted medical and behavioral treatments.

The high test-retest reliability for EEG profiles is present in both TD and ASD groups, though reliability was higher overall in the TD group (ICC 0.858) than the ASD group (ICC 0.807). This is consistent with prior findings suggesting more variable neural activity in ASD compared to TD (David et al., 2016) and may suggest that reliability, in addition to providing important information for biomarker development, may in and of itself represent a potential biomarker. Notably, higher neural variability may reflect (or provoke) more variable emotional states during testing and more variable attention to the stimuli. Such factors are often found to be clinically more variable among children with ASD. Notably, there is also a decrease in ICC of the alpha peak frequency when adjusted for age. This is likely related to the fact that alpha peak frequency typically increases with age; therefore, adjustment for age will absorb some of the across-subject variations, thus making the ratio of across-subject variation to total variation (ICC) decrease. The larger decrease of alpha peak frequency ICC in the TD group with age adjustment may reflect a stronger tendency for alpha peak frequency to increase with age in the TD group as compared to the ASD group; this tendency has been previously described (Edgar et al., 2019). The decrease in alpha bandwidth ICC in the TD group with age adjustment may reflect a similar tendency; however, to our knowledge alpha bandwidth has not been extensively studied in the past, and thus this may be an interesting direction for future studies.

Because the EEG PSD captures a range of parameters, it is important to consider specifically which of those parameters have high short-term test-retest reliability (and thus offer the potential for diagnostic biomarker development), vs. those with low short-term test-retest reliability (potentially reflecting state, attention or perhaps noise). Our findings suggest that within the PSD, a relatively small set of parameters is largely responsible for capturing the fingerprint-like quality of each individual’s EEG. FOOOF-based parameterization suggests that the alpha peak is particularly useful for individualizing the power spectrum. Within the alpha peak, amplitude offers particular promise in this regard, although the center frequency of the alpha peak also provides strong reliability within individuals. Here, it is particularly notable that the frequency of alpha is often considered to be an individual trait (changing only gradually with age and other factors but otherwise remaining relatively stable in most cases), whereas alpha amplitude varies more with the state. For example, the posterior dominant rhythm tends to arise when the eyes are closed and is suppressed with eye-opening; similarly, mu rhythms over the motor cortex are suppressed by imagining or engaging in motor tasks. However, our findings suggest that in the context of the environment in which EEGs were collected in the ABC-CT (watching a silent, screen-saver type videos), alpha amplitude remains quite stable—even more so, in fact, than alpha frequency.

For the slope of the power spectrum as measured by FOOOF, ICC was good in the ASD group but poor in the TD group. This suggests that slope (at least as measured by FOOOF with the parameters used here) is unstable across sessions in the TD group. One possible explanation for this is that the TD group may be more sensitive to session effects (e.g., due to habituation, adaptation, or learning) than the ASD group, and this is being reflected in the slope. It is also possible that the older mean age or lower mean IQ of the ASD group, rather than TD or ASD status *per se*, contributed to this difference. An alternative explanation, supported by a visual review of Figure 2, is that there is very little inter-individual variability in the PSD slope among the TD group; therefore, intra-individual reliability (across days) cannot be much higher than inter-individual reliability (across participants) in the TD group, because inter-individual reliability is high to begin with. In the ASD group, which may be more heterogeneous given the wide variety of genetic and other underlying factors that lead to ASD, the inter-individual variability in slope is higher. In this case, similarly strong intra-individual reliability in the TD and ASD groups would lead to a higher ICC in the ASD group, because of the higher inter-individual variability in this group.

Importantly, the eigenfunctions which best characterized PSD shape exhibited the most variance at relatively low frequencies (4–13 hz), corresponding to overall offsets of the PSD and in the theta to alpha range of the EEG, aligning with the parametric findings from FOOOF and highlighting the import of this frequency range for characterizing stable interindividual differences in brain activity. This finding, combined with the tendency for a variance to be explained by activity at slightly higher frequencies in the ASD group (alpha-beta) than TD participants (predominantly alpha), may help to explain the higher estimated ICC for offset and slope in the ASD group compared to TD. Because the slope and offset terms in FOOOF are fit in the semilog-power space, these parameters are sensitive to power dynamics at higher frequencies, which are often of lower magnitude.

For the nonparametric analyses of relative power, reliability in both groups improves with the removal of peripheral electrodes. Notably, because peripheral electrodes are closer than central electrodes to many non-brain-based sources of detected activity (e.g., muscle and eye movements), they are often more susceptible to artifact than more central electrodes. This suggests (perhaps reassuringly) that brain-based findings, more so than artifact-based findings, remain stable across EEG sessions within an individual. On the other hand, for the parametric analyses of absolute power, the removal of peripheral electrodes does not improve reliability. This may be because the majority of parameters identified by FOOOF are not significantly affected by an artifact in peripheral electrodes, raising the possibility that FOOOF is less susceptible to artifact contamination than nonparametric analyses; this may be further studied in future work.

Nonparametric analyses otherwise reveal complementary results to the parametric analyses. Parametric analyses reveal excellent ICC for the amplitude of the largest alpha peak and good ICC for the frequency of the largest alpha peak. This is true in both the ASD and TD groups, though the ICC in the TD group is slightly higher than that in the ASD group for both of these parameters. Similarly, nonparametric analyses highlight alpha amplitude as capturing the majority of variance for the participant-level spectral densities, followed by alpha frequency. This is again true in both the ASD and TD groups, though slightly more variance is captured by the first two eigenfunctions in the TD as compared to the ASD group. Parametric functions also demonstrate that the dominant peak tended to stay within the alpha band for the TD group, but tended to stay more broadly in the range of both the alpha and beta bands for the ASD group. Similarly, nonparametric functions demonstrate that the TD participants show the largest variation in the alpha band, whereas ASD participants show variation in alpha but also extending into beta.

Nonparametric functional data analysis and FOOOF thus provide convergent and complementary approaches to characterizing the PSD. Nonparametric functional data analysis characterizes PSD shape accurately and with a small number of principal functions yielding high levels of reliability. However, it relies on “learning” these functions based on the current data set and thus yields different principal functions based on the input data, as we see here between our diagnostic groups. Additionally, the resulting functions need careful interpretation to ground their relationship with brain activity. Conversely, FOOOF estimates require more parameters to characterize the PSD. However, fitting these parameters does not depend on the presence of other members of the data set (although the algorithm fitting settings can indirectly force information sharing among power spectra). Also, the interpretation of FOOOF parameters is more direct. FOOOF explicitly attempts to separate biophysically meaningful model parameters such as slope, offset, and oscillatory peaks.

It is important to note the specific questions that the present study is designed to answer. First, the two testing days for each individual took place within approximately a week. While this suggests promise for biomarker development in trials where EEG-based findings are expected to change over very short periods, many pharmacological interventions aim to change neural activity over the longer term (weeks, months, or longer). Examining test-retest stability of the EEG power spectrum over these longer periods is part of ongoing analyses for the ABC-CT main study, which will include 6 weeks and 6-month follow-up recordings. Additionally, here we report only test-retest reliability for a single set of EEG measures, all based on the power spectrum. EEG is a rich source of information beyond that which can be captured in the power spectrum, in both the time domain and the frequency domain. As future studies suggest additional EEG-based measurements that may offer promise for biomarker developments, the test-retest reliability of the measurements will need to be explicitly evaluated. Finally, the data presented here specifically evaluates ICC and group variability thereof (ASD vs. TD); however, our sample size was not large enough to compare ICC across sites. Other analyses relevant to the EEG power (e.g., comparing power, rather than ICC thereof, across groups) are underway for the larger “Main Study” of ABC-CT but are beyond the scope of the data presented here.

Developing biomarkers for ASD and other neurodevelopmental disorders remains a high priority in the field, given the potential benefits, biomarkers offer for clinical trials, diagnostics, and monitoring (Krueger et al., 2017). While future studies will continue to assess which measurements (in EEG and otherwise) offer the most promise as potential biomarkers of various types, our findings of high short-term test-retest reliability of the EEG power spectral density are a crucial step towards ensuring that potential biomarkers meet necessary criteria for validation.

## Data Availability Statement

Datasets analyzed for this study can be found in the National Database for Autism Research https://ndar.nih.gov/ - #2288.

## Ethics Statement

The studies involving human participants were reviewed and approved by central Institutional Review Board at Yale University. Written informed consent to participate in this study was provided by the participants’ parent/legal guardian.

## Author Contributions

AL, AN, AS, SW, FS, CS, MM, RB, KC, GD, SF, SJ, CN, JM, and DŞ made substantial contributions to the conception or design of the ABC-CT and provided critical revisions related to the important intellectual content. AL, AN, AS, and DŞ contributed to the analysis of the data described in this manuscript. AL, AN, AS, SW, and DŞ contributed to the drafting of this manuscript. All named authors read and provided approval for publication of the content.

## Conflict of Interest

AL, AN, AS, SW, CS, MM, RB, KC, SF, CN, and DŞ declare that the research was conducted in the absence of any commercial or financial relationships that could be construed as a potential conflict of interest. FS is a consultant for and has received research funding from both Janssen Research and Development and Roche Pharmaceutical Company. GD is on the Scientific Advisory Boards of Janssen Research and Development, Akili, Inc., LabCorp, Inc., Tris Pharma, and Roche Pharmaceutical Company, a consultant for Apple, Inc, Gerson Lehrman Group, Guidepoint, Inc., Teva Pharmaceuticals, and Axial Ventures, has received grant funding from Janssen Research and Development, and is CEO of DASIO, LLC. Dawson has developed technology that has been licensed and Dawson and Duke University have benefited financially. Dawson receives royalties from Guilford Press, Springer, and Oxford University Press. SJ is a consultant for Roche Pharmaceutical Company, and receives grant funding from Roche Pharmaceutical Company. JM consults with BlackThorn Therapeutics, has received research funding from Janssen Research and Development, and receives royalties from Guilford Press, Lambert, and Springer. The reviewer SA declared a shared affiliation, with no collaboration, with several of the authors, [AL, SF, CN], to the handling editor at the time of review.

## Footnotes

^1^www.asdbiomarkers.org
