# Supplementary material for: Day-to-Day Test-Retest Reliability of EEG Profiles in Children With Autism Spectrum Disorder and Typical Development
Source: Front Integr Neurosci. 2020 Apr 30;14:21. doi: 10.3389/fnint.2020.00021 (PMC7204836; doi:10.3389/fnint.2020.00021)
Supplement: Supplementary file 1 [file Data_Sheet_1.DOCX]

Supplementary Material

**Supplementary Table 1:** The age-adjusted estimated intraclass correlation coefficients (ICCs) and their 95% bootstrap confidence intervals for the six FOOOF parameters for each diagnostic group.

| FOOOF Parameter | TD | ASD |
| --- | --- | --- |
| Offset | 0.420 (0, 0.752) | 0.464 (0.093, 0.708) |
| Slope | 0.225 (0, 0.627) | 0.687 (0.277, 0.81) |
| Number of peaks | 0.021 (0, 0.49) | 0.248 (0, 0.578) |
| Largest alpha peak: Center Frequency | 0.494 (0.11, 0.766) | 0.482 (0.039, 0.77) |
| Largest alpha peak: Amplitude | 0.866 (0.727, 0.94) | 0.835 (0.659, 0.926) |
| Largest alpha peak: Bandwidth | 0.256 (0, 0.571) | 0.355 (0.053, 0.717) |

**Supplementary Table 2:** The IQ-adjusted estimated intraclass correlation coefficients (ICCs) and their 95% bootstrap confidence intervals for the six FOOOF parameters for each diagnostic group.

| FOOOF Parameter | TD | ASD |
| --- | --- | --- |
| Offset | 0.499 (0.006, 0.759) | 0.516 (0.102, 0.761) |
| Slope | 0.298 (0, 0.694) | 0.712 (0.433, 0.814) |
| Number of peaks | 0.021 (0, 0.513) | 0.154 (0, 0.593) |
| Largest alpha peak: Center Frequency | 0.71 (0.38, 0.882) | 0.584 (0.368, 0.815) |
| Largest alpha peak: Amplitude | 0.847 (0.641, 0.926) | 0.812 (0.608, 0.919) |
| Largest alpha peak: Bandwidth | 0.44 (0, 0.644) | 0.344 (0.046, 0.704) |
